# Biomarkers of Intrathecal Synthesis May Be Associated with Cognitive Impairment at MS Diagnosis

**DOI:** 10.3390/ijms26020826

**Published:** 2025-01-19

**Authors:** Eleonora Virgilio, Valentina Ciampana, Chiara Puricelli, Paola Naldi, Angelo Bianchi, Umberto Dianzani, Domizia Vecchio, Cristoforo Comi

**Affiliations:** 1 Neurology Unit, Department of Clinical and Biological Sciences, University Hospital San Luigi Gonzaga, University of Turin, 10043 Turin, Italy; eleonora.virgilio@unito.it; 2 Interdisciplinary Research Center of Autoimmune Diseases (IRCAD), Department of Health Sciences, University of Piemonte Orientale, 28100 Novara, Italy; umberto.dianzani@maggioreosp.novara.it (U.D.); domizia.vecchio@gmail.com (D.V.); 3 Neurology Unit, Department of Translational Medicine, University Hospital Maggiore della Carità di Novara, University of Piemonte Orientale, 28100 Novara, Italy; 20022116@studenti.uniupo.it (V.C.); pnaldi@inwind.it (P.N.); bianchi.angelo.94@gmail.com (A.B.); 4 Clinical Biochemistry, University Hospital Maggiore della Carità di Novara, University of Piemonte Orientale, 28100 Novara, Italy; 20032501@studenti.uniupo.it

**Keywords:** multiple sclerosis, biomarker, kappa index, lambda index, kappa free light chain, oligoclonal band, cognition

## Abstract

The pathophysiology of cognitive impairment (CI) in multiple sclerosis (MS) remains unclear. Meningeal B cell aggregates may contribute to cortical grey matter pathology. Cerebrospinal fluid (CSF), kappa free light chains (KFLC), and KFLCs-Index (kappa-Index) are reliable quantitative markers of intrathecal synthesis, but few data have been presented exploring the association with CI, and no data are present for lambda FLC (LFLC) in MS. We evaluated cognition using the Brief International Cognitive Assessment for MS (BICAMS) battery and collected serum and CSF at diagnosis in newly diagnosed drug-naïve MS patients. We observed that patients with impaired verbal memory and overall CI showed increased CSF KFLCs (respectively *p*: 0.0003 and *p*: 0.003) and kappa-Index (respectively *p*: 0.01 and *p*: 0.02) compared to those with normal verbal memory and no CI. Patients with CI also displayed lower CSF LFLCs (*p*: 0.04) and lambda-Index (*p*: 0.001); however, only CSF KFLC negatively correlated with normalized results of verbal memory (for age, sex, and educational levels), even after correction for EDSS (r: −0.27 *p*: 0.01). Finally, CSF FKLC and kappa-Index were significant predictors of verbal memory in a multivariate analysis. Our results, suggest that intrathecal B cell activity might contribute to CI development in MS patients.

## 1. Introduction

Multiple sclerosis (MS) is an inflammatory immune-mediated central nervous system disease. Patients can experience cognitive difficulties from early disease stages, and cognition might be affected in several domains, mainly information processing speed (IPS), verbal memory (VM), visuospatial memory (VSM), and verbal fluency [1]. Although cognitive symptoms might be difficult to intercept at diagnosis, their late impact on quality of life is relevant, and efficacy data of disease-modifying therapies are conflicting on these parameters [1]. Moreover, the pathophysiology of cognitive impairment (CI) in MS remains unclear. Cortical grey matter (GM) pathology seems to be involved in this process and a role may be played by ectopic meningeal B cell aggregates [2], but no reliable specific fluid biomarkers are available for CI in MS.

Cerebrospinal fluid (CSF) analysis is a complementary test in MS diagnosis [3] for demonstrating intrathecal immunoglobulin (Ig) synthesis evaluated with a qualitative or quantitative approach as markers of intrathecal B cell activity. The most reliable qualitative approach is isoelectrofocusing [4], which shows the presence of oligoclonal bands (OCBs) in 90% of MS patients [5,6]. OCBs are the gold standard for IgG intrathecal synthesis determination, and their diagnostic and prognostic value is well established. Patients with OCBs convert easily to clinically defined MS, and MS patients with OCBs have a poor motor prognosis [7]. However, although OCBs allow an early diagnosis and have a negative prognostic value, their possible involvement in cognition is poorly investigated. Farina et al. showed that OCBs at MS diagnosis were associated with increased GM pathology, physical disability, and CI after 10 years. Moreover, the CSF of OCB-positive patients display high levels of cytokines, marking B cell activation, lymphoid-neogenesis, and pro-inflammatory immune response, which supports the crucial role played by compartmentalized, intrathecal B cell response in the pathogenesis of the cortical lesions, CI, and intrathecal synthesis production [7]. Quantitative indexes of intrathecal IgG production such as the Link Index (ratio of serum and CSF IgG and albumin in serum and CSF) can also be used [3]. The Link Index is >0.7 in 70–90% of MS patients. Other intrathecal synthesis markers have recently been introduced [8,9], such as CSF kappa free light chains (KFLCs) and the kappa-Index. They have proved to be a reliable diagnostic marker of intrathecal synthesis [8,9], whereas lambda FLCs (LFLC) displayed less consistent diagnostic results [10,11]. Similarly to OCBs, the prognostic role of KFLC has been examined, with a particular focus on inflammatory activity and motor outcomes [12,13,14,15,16,17,18], and only two studies investigated cognition [19,20]. Those recent findings suggested a possible role of kappa-Index in the development of impaired IPS and VM [19,20], whereas no data are present for LFLC, lambda-Index, and cognition specifically in MS patients. In this study, we aimed to explore the correlation between several B cell activity biomarkers and CI in MS drug-naïve patients at diagnosis.

## 2. Results

Baseline characteristics are reported in Table 1. We enrolled a young cohort of patients (mean age at Diagnosis of 37.7 ± 10.6 years) with a mean educational level of 13.26 years and a low median expanded disability status score (EDSS) at diagnosis (1.5 ranging from 0–4): 91% were relapsing-remitting MS (RRMS), and the remaining 9% were progressive MS. Concerning MRI characteristics, most of them presented high lesion load and dissemination in the spinal cord, but only 38.5% presented at least one gadolinium-enhancing (gd+) lesion. Most of them (83.3%) had OCBs. The mean values of CSF KFLC were 0.61 ± 0.61 mg/dL, CSF LFC 0.26 ± 0.64 mg/dL, Link Index 0.87 ± 0.51, lambda-Index 19.72 ± 24.51 (median 9.36, range 1.35–138.3), and kappa-Index 79.6 ± 86.52 (median 47.50, range 2.0–449.8), with only 2 patients (2.5%) ≤2.4 and 20 patients (25.6%) ≤100.

Results from the BICAMS battery are summarized in Table 2. The most altered test was the California verbal learning test-2 (CVLT-2), corresponding to an alteration of the VM (T-scores ≤ 35 in 22% of the cohort). Symbol Digit Modalities Test (SMDT) was impaired in fifteen (19%) patients, whereas only nine (12%) patients were impaired at Brief Visuospatial Memory Test–Revised (BVMT-R). Overall, nine patients showed a composite T-score below normality (12%), whereas twenty-nine (37%) patients showed impairment at least in one test, eleven (14%) at two tests, and only three (4%) failed in all three test batteries.

Patients with or without CI did not differ for gender (*p*: 0.8), EDSS (median of 1.5 vs. 1.2 *p*: 0.2), age at onset (37.5 ± 10.6 vs. 33.8 ± 9.3 years old *p*: 0.2), or age at diagnosis (40.24 ± 10.9 vs. 36.18 ± 10.2 *p*: 0.1). The only significant difference was the educational level: patients with CI displayed a lower education than those without CI (14.3 ± 3.3 education years vs. 11.5 ± 3.0, *p*: 0.0008). Patients with higher lesion load at baseline displayed lower composite T-scores compared with those with a low white matter lesion load (WMLL low vs. high 49.45 ± 9.52 vs. 44.46 ± 8.42 *p*: 0.028), whereas no differences were observed for gd+ lesions (no vs. yes 45.45 ± 9.78 vs. 47.7 ± 7.88 *p*: 0.2) or spinal lesions (yes vs. no 46.34 ± 7.7 vs. 46.26 ± 11.41 *p*: 0.8).

**Figure 1:**
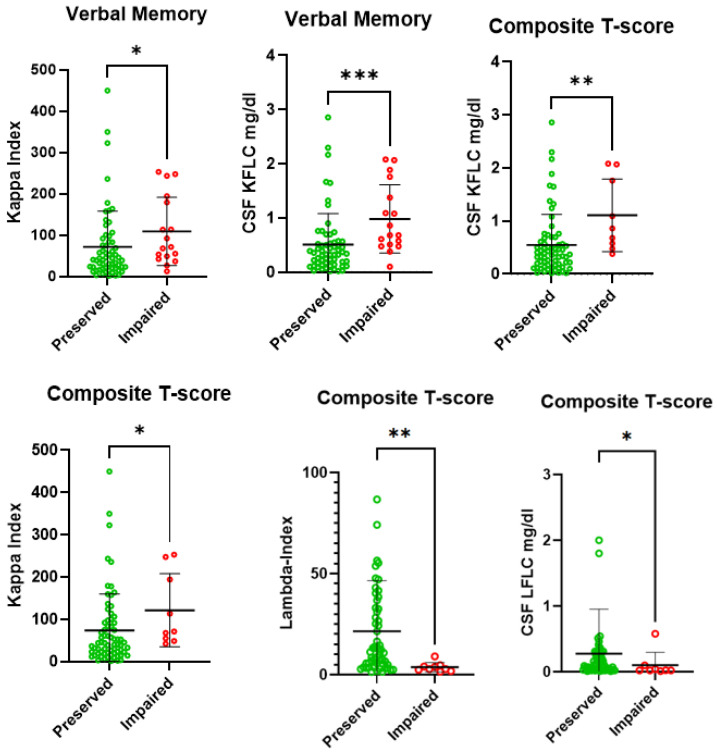
Intrathecal synthesis biomarkers and cognitive impairment. Significant differences in intrathecal synthesis fluid biomarkers in MS patients with and without impairment in verbal memory and in overall cognition are represented (single values, mean and SD). Abbreviations: CSF = cerebrospinal fluid, KFLC = kappa free light chain. *: *p* ≤ 0.05, **: *p* ≤ 0.01 and ***: *p* ≤ 0.001.

Patients with impaired VM and CI (particularly at the composite T-score) showed increased CSF KFLCs (respectively *p*: 0.0003 and *p*: 0.003) and kappa-Index (respectively *p*: 0.01 and *p*: 0.02) compared to those with normal VM and no CI, as reported in Table 3 and Figure 1, whereas patients with lower CSF LFLC and lambda-Index displayed lower composite scores (*p*: 0.04 and 0.001 Table 3 and Figure 1).

Patients with OCBs showed a trend of higher kappa-Index compared to those without OCBs (mean values 85.88 ± 90.79 vs. 48.20 ± 52.88 *p*: 0.06), whereas no differences were observed for lambda-Index (mean values 19.33 ± 25.63 vs. 21.61 ± 10.06 *p*: 0.5). No gender or age differences were observed for kappa-Index, lambda-Index, Link-Index, and OCBs (*p* > 0.05). No biomarker differences were observed when stratifying patients according to MRI characteristics based on high and low WMLL and spinal cord lesions. Interestingly, CSF KFLC and kappa-Index were higher in patients with a gd+ lesion (*p*: 0.03 and *p*: 0.01 Table 3), whereas no differences were observed for LFLC, Link-Index, and OCBs.

Several fluid intrathecal synthesis biomarkers were inversely correlated with either a single or multiple cognitive tests (Table 4).

The most significant, although mild, correlation was between CSF KFLCs and normalized CVLT2 T-scores (r: −0.37, *p*: 0.001) as well as composite T-score (r: −0.30 *p*: 0.007); kappa-Index and Link Index also showed similar results, although with a weaker correlation (Table 4). CSF KFLC was also inversely correlated with BVMT-R scores, whereas kappa-Index and Link were not. Even though patients with CI displayed lower CSF LFLCs (*p*: 0.04) and lambda-Index (*p*: 0.001), no correlation was observed in the univariate model (Table 4).

In addition, OCBs did not correlate with cognitive performance (*p* > 0.05). The inverse correlation between CSF KFLC and CVLT2 T-score (r: −0.27 *p*: 0.01) and composite T-score (r: −0.22 *p*: 0.048) was also confirmed after correction for EDSS.

Previous studies hypothesized that a cut-off of kappa-Index >100 at baseline had a twice as high probability of a second clinical attack within 12 months [15], and kappa-Index ≥106 detects patients at increased risk of relapse [14]. Recently, Rosenstein et al. observed [20] that patients with kappa-Index >100 showed reduced SDMT raw scores at follow-up, compared with their baseline scores. In our cohort, no correlation was observed between EDSS at diagnosis and kappa-Index (*p*: 0.4), but patients with kappa-Index >100 (N:20) showed lower values of Composite T-Scores (43.26 ± 7.20 vs. 47.37 ± 9.51 *p*: 0.05); however, no differences were observed for single cognitive tests, particularly SDMT (*p* > 0.05).

Based on univariate analysis, we performed a multivariate analysis using CSF KFLC, CSF LFLC, kappa-Index, and lambda-Index along with EDSS, age, gender, and MRI characteristics as independent variables and SDMT T-scores, CLVT2 T-Scores, BVMT-R T-scores, and Composite T-scores as dependent variables. The only model reaching a global *p* < 0.05 was the VM model where CSF KFLC (beta −0.968, *p*: 0.005, 95% CI −30.099–−5.593) and kappa-Index (beta 0.854, *p*: 0.017 95% CI 0.021–0.207) were significant predictors of CLVT2 T-scores (shown in Supplementary Materials Table S1).

## 3. Discussion

CSF is an important diagnostic and prognostic tool in MS management. Recently, measures of FLC (easily detected by nephelometry) have been proposed as an alternative method to identify intrathecal synthesis [3,8,9,11]. The presence and the number of IgG OCBs [21] were identified as a negative prognostic factor to consider at diagnosis [22], as well as IgM OCBs [23]. Some evidence supports the predictive value of KFLC on motor evolution, treatment failure, occurrence of relapses, and progression, independent of inflammatory activity [12,13,15,16,17,18,24,25], but little is known about the contribution to cognition [19,20]. Solid evidence showed a high diagnostic accuracy of intrathecal KFLC synthesis in MS with a sensitivity and specificity of approximately 90%, similar to OCBs [3,8]. Nonetheless, KFLCs have advantages as their detection is easy, fast, reliable, cost-effective, and rater independent. They also return quantitative results, which might also improve the value of predicting MS disease activity [8,9].

Our study highlights that CSF KFLCs and kappa-Index are increased in patients with gd+ lesions, supporting the role of B cells during acute disease flare [26,27]. Interestingly, in this study, patients with kappa-Index >100 show decreased cognitive performances (with the Composite T-Scores) and, crossectionally, CSF KFLC and kappa-Index are elevated and negatively correlated (although mildly) in patients with impaired VM and CI at diagnosis. A weak correlation is also present when considering normalized T-scores corrected for age, gender, and educational levels, and after correcting for EDSS. In addition, CSF KFLC, and kappa-Index are the only significant predictors of VM performances among MRI and clinical characteristics. However, no similar results were obtained when considering global cognition or other cognitive domains, thus reducing the soundness of our results.

Few data are reported in the literature on the correlation between cognition and fluid biomarkers, mainly neurofilaments light chains and vitamin D [28,29,30]. However, the potential pathogenic role of B cells in CI development in the disease has been suggested by studies focusing on the association with other biomarkers of B cell activation. One study evaluated long-term cognition and baseline OCB status [7], observing that patients with OCBs displayed increased cortical lesions and decreased cognitive performances with the Rao brief repeatable battery. Another study, using an extensive neuropsychological battery, showed that OCB-positive patients had decreased visual–spatial memory performances [31]. Recently, two studies have focused on the quantitative markers of B-cell activation and cognition [19,20]. First, a longitudinal retrospective cohort study on 77 RRMS patients using the SMDT observed that high kappa-Index was associated with a decreased IPS (using the ≥8 points cut-off reduction) over time compared with their baseline [19]. Second, consistently with our results, Gaetani et al. observed in a group of 39 MS patients that kappa-Index negatively correlated with verbal learning and memory, independently of age, disease duration, EDSS, and brain lesion load [20].

Of note, we also investigated for the first time cognition and LFLC, lambda-index in MS patients, observing that patients with CI displayed decreased CSF LFLCs and lambda-Index. Less consistent data are available for LFLC in the literature; some studies report higher levels of CSF LFLC and lambda-index in MS patients, although to a lesser extent compared with kappa-Index [18,32,33], whereas others not [10,11]. Additionally, in the literature, LFLC showed lower diagnostic accuracy compared with KFLC [33]. Finally, no data are available for cognition except for one recent study in Alzheimer’s disease patients [34].

Our data support the usefulness of B-cell markers at MS diagnosis, possibly indicating a weak relationship between CSF KFLC and kappa-Index with VM, although further analysis is needed to confirm or exclude this association. To our knowledge, this is the first study to include serum and CSF LFLC, KFLC, and cognition with the BICAMS using raw scores and normalized scores for age, sex, and educational levels. Our analysis has several limitations. Firstly, the small sample size and inclusion of both progressive (although a minority) and relapsing-remitting patients. However, patients were clinically well characterized, displayed a low disability and were drug naïve. Secondly, the lack of fatigue and depression scales could affect cognitive results, even though we excluded patients in need of psychiatric treatments. Finally, further limits are the cross-sectional nature of the study and the lack of prospective data on cognition (for the evaluation of cognitive trajectories over time) and disability over time.

## 4. Materials and Methods

### 4.1. Study Population

This monocentric study enrolled 78 newly diagnosed MS patients from 2015 to 2022. We selected patients who performed lumbar puncture (LP) as part of the usual diagnostic MS work-up. We enrolled patients with a diagnosis of MS according to Mc Donald criteria 2010 or the 2017 revision [35], aged at least 18 years old, who signed an informed consent form for both diagnostic and research purposes at the moment of LP, and with a cognitive evaluation routinely performed in the same setting, or within one month from baseline. We excluded patients with a history of psychiatric diseases, those treated with psychoactive drugs, alcohol abusers, and patients previously treated with immunosuppressants. Patients were not exposed to steroids during LP or cognitive evaluation. We collected clinical demographic data such as gender, age of onset, age at diagnosis, MS phenotype, and EDSS at diagnosis. According to Italian guidelines, brain and spinal MRIs were performed within three months before or following baseline [36]. We recorded T2 WMLL with a cut-off of ten lesions to discriminate high from low lesion load [37] and the presence or absence of spinal lesions and/or gd+ lesions.

### 4.2. Neuropsycological Evaluation

Cognition at baseline was checked using the BICAMS battery. This battery, largely used in clinical practice and research settings, is formed by three tests: the SDMT for IPS, the CVLT2 for and VM, and the BVMT-R for VSM. According to the Italian normative values, raw scores were corrected for educational level, age, and gender. Regression-based T-scores were thus obtained [38]. A composite T-score was also calculated as the mean of the three single normalized scores of the patient. The presence of a specific cognitive domain impairment was defined by the failure of the corresponding test (T-score ≤ 35) [38]. CI was characterized by impairment in at least 1/3 test and/or a composite T-score ≤ 35.

### 4.3. Serum and CSF Analysis

Matched CSF and serum samples were obtained and consecutively analyzed during the diagnostic work-up. Every patient was tested for cell counts, glucose, and protein CSF concentration, OCBs were detected via isoelectrofocusing (Sebia), while serum and CSF albumin, LFLCs, and KFLCs were measured via nephelometry (BN II System by Siemens, Munich, Germany) [3,11]. Kappa-Index and lambda-Index correspond to the ratio between CSF/serum KFLC or LFLC and CSF/serum albumin. The serum and CSF analysis were performed by board-certified laboratory technicians blinded to the patient’s clinical status. All analyses were performed in the University of Piemonte Orientale Clinical Biochemistry Laboratory in Novara, Italy.

### 4.4. Statistical Analysis

We used SPSS 25.0 (SPSS Inc., Chicago, IL, USA) and Graphpad Prism 10 for Windows (La Jolla, CA, USA) for statistical analysis. We presented categorical data with median, range, and interquartile range (IQR), proportions as numbers and percentages, and continuous data with mean and standard deviation (SD). Normal distribution was checked based on the results of the Shapiro–Wilk test. An exploratory analysis was performed comparing biomarker values to clinical and MRI characteristics. The *t*-test was used for normally distributed variables, whereas the Mann–Whitney U test and the Kruskal–Wallis test were used for non-normally distributed continuous variables, and Chi-Squared test and Fisher test for categorical variables. Spearman’s rank correlation coefficient test was used to determine the correlation between continuous variables. Linear regression analyses were performed with biomarkers, clinical, and MRI characteristics at baseline as independent variables and cognitive parameters as dependent variables. All tests were two-sided, and the significance threshold was set to *p* < 0.05.

### 4.5. Ethical Approval

Upon CSF sampling, patients gave written consent to CSF storage for research purposes. Plus, the study was conducted following the declaration of Helsinki guidelines and approved by the Ethical Committee of the University Hospital of Novara (CE262/2022). Collected data were used to produce a pseudonymized dataset.

## 5. Conclusions

Our results expand knowledge on the possible role of B cells in the pathogenesis of CI, particularly VM in MS patients. Further studies are needed to explore this association.

## Figures and Tables

**Table 1:** Demographic, clinical, MRI characteristics, and CSF mean levels of biomarkers of the study population.

Demographic Characteristics	
Age at onset (yrs); mean ± SD	35.1 ± 9.9
Age at Diagnosis (yrs); mean ± SD	37.7 ± 10.6
Female n, (%)	52 (66.7%)
Educational level (yrs) mean ± SD	13.26 ± 3.53
EDSS at diagnosis; median; (range)	1.5 (0–4)
**MS Type (n; %)**	
Relapsing MS	71 (91%)
Progressive MS	7 (9%)
**MRI characteristics (n; %)**	
WMLL > 9 T2 brain lesions	49 (63%)
WMLL ≤ 9 T2 brain lesions	29 (37%)
Gd+ lesions	30 (38.5%)
Spinal lesions	51 (64%)
**Biomarker (mean ± SD)**	
CSF OCB Pattern 2, n %	65 (83.3%)
CSF KFLC (mg/dL)	0.61 ± 0.61
CSF LFLC (mg/dL)	0.26 ± 0.64
Serum KFLC (mg/dL)	1.54 ± 0.54
Serum LFLC (mg/dL)	1.46 ± 0.60
Kappa-Index (mean ± SD)	79.60 ± 86.52
Lambda-Index (mean ± SD)	19.72 ± 24.51
Link Index	0.87 ± 0.51

Abbreviations: CSF = cerebrospinal fluid, EDSS = expanded disability status score, Gd+ = gadolinium enhancing, OCB = oligoclonal bands, KFLC = kappa free light chains, LFLC = lambda free light chains, SD = standard deviation, yrs = years.

**Table 2:** Neuropsychological results at the BICAMS evaluation.

Cognitive Domain	Test	Raw Score (Mean ± SD)	T-Score (Mean ± SD)	Score < Cut-Off (n; %)
**IPS** **VM** **VSM** **Overall cognition** **Cognitive Impairment** **1/3 tests < cut-off** **2/3 tests < cut-off** **3/3 tests < cut-off**	SDMT CVLT-II BVMT-R Composite T-score	50.60 ± 13.47 52 ± 11.58 24.60 ± 8.39 NA	46.33 ± 11.79 45.78 ± 11.65 46.83 ± 10.95 46.31 ± 9.11	15/78; 19% 17/78; 22% 9/78; 11% 9/78; 11% 29/78; 37% 11/78 (14%) 3/78 (4%)

Results are presented as raw scores for each test and normalized T-scores for each test. Number and percentage of the altered test (normalized score below cut-off) are also shown. Cognitive impairment was defined as the presence of at least one out of the three tests below the normality cut-off. Overall composite score was calculated as a mean of the single normalized scores. Abbreviations: BVMT-R = brief visuospatial memory revised test, CVLT-II = California verbal learning test-II, IPS = information processing speed, SDMT = symbol digit modalities test, SD = standard deviation, NA = not available, VM = verbal memory, VSM = visuospatial memory.

**Table 3:** Comparison between biomarkers, cognition, and radiological characteristics.

		**CSF KFLC**		**Kappa-Index**		**Link-Index**	
		**Mean**	***p*** **-Value**	**Mean**	***p*** **-Value**	**Mean**	***p*** **-Value**
**IPS**	Impaired (N: 15) Not impaired (N: 63)	0.61 ± 0.57 0.61 ± 0.62	0.8	84.88 ± 92.06 78.34 ± 85.88	0.8	0.93 ± 0.58 1.21 ± 2.88	0.7
**VM**	Impaired (N17) Not impaired (N61)	0.98 ± 0.63 0.50 ± 0.57	***0.0003***	109.10 ± 82.53 71.39 ± 86.47	***0.01***	1.03 ± 0.52 1.2 ± 2.9	0.08
**VSM**	Impaired (N: 9) Not impaired (N: 69)	0.65 ± 0.56 0.60 ± 0.62	0.7	69.27 ± 69.73 80.95 ± 88.82	0.8	0.87 ± 0.51 1.20 ± 2.75	0.9
**CI** **(≥1 test ≠)**	Impaired (N: 29) Not impaired (N: 49)	0.79 ± 0.62 0.50 ± 0.58	***0.01***	96.09 ± 89.34 69.84 ± 84.22	0.09	0.98 ± 0.56 1.27 ± 3.26	0.2
**Overall** **Composite**	Impaired (N: 9) Not Impaired (N: 69)	1.11 ± 0.68 0.54 ± 0.57	***0.003***	121.6 ± 86.53 74.12 ± 85.63	***0.02***	1.04 ± 0.48 1.17 ± 2.75	0.1
**GD lesion**	Present (N: 30) Absent (N: 48)	0.73 ± 0.70 0.40 ± 0.37	***0.03***	97.95 ± 98.49 50.23 ± 52.03	***0.01***	1.6 ± 4.1 0.8 ± 0.5	0.8
**Spinal**	Present (N: 51) Absent (N: 27)	0.64 ± 0.66 0.54 ± 0.50	0.6	83.51 ± 95.44 72.21 ± 67.59	0.9	0.89 ± 0.57 1.66 ± 4.31	0.4
**WMLL**	High (N: 49) Low (N: 29)	0.62 ± 0.56 0.58 ± 0.69	0.3	74.38 ± 66.77 88.41 ± 113.20	0.5	1.33 ± 3.22 0.85 ± 0.56	0.3
		**Serum KFLC**		**Serum LFLC**		**CSF LFLC**	
		**Mean**	***p*** **-Value**	**Mean**	***p*** **-Value**	**Mean**	***p*** **-Value**
**IPS**	Impaired (N: 15) Not impaired (N: 63)	1.38 ± 0.43 1.57 ± 0.55	0.4	1.36 ± 0.55 1.48 ± 0.61	0.8	25.11 ± 40.12 18.47 ± 19.66	0.4
**VM**	Impaired (N17) Not impaired (N61)	1.53 ± 0.36 1.53 ± 0.57	0.6	1.58 ± 0.42 1.43 ± 0.64	0.1	16.33 ± 21.55 20.58 ± 25.33	0.9
**VSM**	Impaired (N: 9) Not impaired (N: 69)	1.47 ± 0.29 1.54 ± 0.56	0.8	1.42 ± 0.48 1.46 ± 0.61	0.6	6.49 ± 4.0 21.09 ± 25.34	0.5
**CI** **(≥1 test ≠)**	Impaired (N: 29) Not impaired (N: 49)	1.41 ± 0.39 1.60 ± 0.59	0.3	1.40 ± 0.47 1.49 ± 0.66	0.8	22.72 ± 32.08 18.03 ± 19.28	0.9
**Overall** **Composite**	Impaired (N: 9) Not Impaired (N: 69)	1.49 ± 0.40 1.54 ± 0.55	0.7	1.57 ± 0.44 1.45 ± 0.61	0.4	2.87 ± 1.23 21.46 ± 25.12	***0.04***
**GD lesion**	Present (N: 30) Absent (N: 48)	1.55 ± 0.49 1.52 ± 0.56	0.9	1.53 ± 0.75 1.41 ± 0.48	0.8	21.63 ± 20.91 18.49 ± 26.75	0.3
**Spinal**	Present (N: 51) Absent (N: 27)	1.48 ± 0.42 1.64 ± 0.70	0.4	1.41 ± 0.41 1.56 ± 0.87	0.7	20.02 ± 26.80 19.10 ± 19.56	0.2
**WMLL**	High (N: 49) Low (N: 29)	1.49 ± 0.46 1.61 ± 0.64	0.5	1.39 ± 0.43 1.55 ± 0.77	0.3	18.05 ± 19.52 22.01 ± 30.31	0.4
		**Lambda-Index**					
		**Mean**	***p*** **-Value**				
**IPS**	Impaired (N: 15) Not impaired (N: 63)	25.11 ± 40.12 18.47 ± 19.66	0.5				
**VM**	Impaired (N17) Not impaired (N61)	16.33 ± 21.55 20.58 ± 25.33	0.3				
**VSM**	Impaired (N: 9) Not impaired (N: 69)	6.49 ± 4.0 21.09 ± 25.34	0.2				
**CI** **(≥1 test ≠)**	Impaired (N: 29) Not impaired (N: 49)	22.72 ± 32.08 18.03 ± 19.28	0.9				
**Overall** **Composite**	Impaired (N: 9) Not Impaired (N: 69)	2.87 ± 1.23 21.46 ± 25.12	***0.001***				
**GD lesion**	Present (N: 30) Absent (N: 48)	21.63 ± 20.91 18.49 ± 26.75	0.2				
**Spinal**	Present (N: 51) Absent (N: 27)	20.02 ± 26.80 19.10 ± 19.56	0.4				
**WMLL**	High (N: 49) Low (N: 29)	18.05 ± 19.52 22.01 ± 30.31	0.6				

Patients are stratified based on normalized T-scores for each test or on radiological characteristics. Biomarkers are expressed either in mg/dL or index. Cognitive impairment was defined as the presence of at least one out of the three tests below the normality cut-off. Overall composite score was calculated as a mean of the single normalized scores. Comparisons are made using either *t*-test or Mann–Whitney U test and Kruskal–Wallis when appropriate. Statistically significant p-value are represented in bold/italics. Abbreviations: CI = cognitive impairment, CSF = cerebrospinal fluid, GD = gadolinium, IPS = information processing speed, KFLC = kappa free light chains, LFLC = lambda free light chains, VM = verbal memory, VSM = visuospatial memory, WMLL = white matter lesion load, ≠: altered.

**Table 4:** Correlation analysis between CSF biomarkers and cognition.

Biomarker	Test	R	*p* Value
CSF KFLC mg/dL	SDMT Raw Scores	−0.19	0.09
	SDMT T-Scores	−0.16	0.1
	CVLT2 Raw Scores	−0.28	***0.01***
	CVLT2 T-Scores	−0.37	***0.001***
	BVMTR Raw Scores	−0.30	***0.007***
	BVMTR T-Scores	−0.23	***0.044***
	Composite T-Score	−0.30	***0.007***
Kappa-Index	SDMT Raw Scores	−0.14	0.2
	SDMT T-Scores	−0.20	***0.08***
	CVLT2 Raw Scores	−0.12	0.2
	CVLT2 T-Scores	−0.25	***0.02***
	BVMTR Raw Scores	−0.19	0.09
	BVMTR T-Scores	−0.15	0.1
	Composite T-Score	−0.23	***0.044***
Link Index	SDMT Raw Scores	−0.1	0.3
	SDMT T-Scores	−0.16	0.1
	CVLT2 Raw Scores	−0.15	0.1
	CVLT2 T-Scores	−0.30	***0.007***
	BVMTR Raw Scores	−0.21	0.07
	BVMTR T-Scores	−0.21	0.06
	Composite T-Score	−0.21	***0.048***
serum KFLC mg/dL	SDMT Raw Scores	No correlation	ns
	SDMT T-Scores		
	CVLT2 Raw Scores		
	CVLT2 T-Scores		
	BVMTR Raw Scores		
	BVMTR T-Scores		
	Composite T-Score		
CSF LFLC mg/dL	SDMT Raw Scores	No correlation	ns
	SDMT T-Scores		
	CVLT2 Raw Scores		
	CVLT2 T-Scores		
	BVMTR Raw Scores		
	BVMTR T-Scores		
	Composite T-Score		
serum LFLC mg/dL	SDMT Raw Scores	No correlation	ns
	SDMT T-Scores		
	CVLT2 Raw Scores		
	CVLT2 T-Scores		
	BVMTR Raw Scores		
	BVMTR T-Scores		
	Composite T-Score		
Lambda-Index	SDMT Raw Scores	No correlation	ns
	SDMT T-Scores		
	CVLT2 Raw Scores		
	CVLT2 T-Scores		
	BVMTR Raw Scores		
	BVMTR T-Scores		
	Composite T-Score		

Univariate analysis was performed with Spearman’s rank correlation coefficient test. Abbreviations: CSF = cerebrospinal fluid, KFLC = kappa free light chains, LFLC = lambda free light chains, BVMT-R = brief visuospatial memory revised test, CVLT2 = California verbal learning test-2, SDMT = symbol digit modalities test. Statistically significant *p*-value are represented in bold/italics.

## Data Availability

The data presented in this study are available on request from the corresponding author. The data are not publicly available due to privacy reasons.

## Supplementary Materials

The supporting information can be downloaded at: https://www.mdpi.com/article/10.3390/ijms26020826/s1.
