# Supplementary material for: Biomarkers of Intrathecal Synthesis May Be Associated with Cognitive Impairment at MS Diagnosis
Source: Int J Mol Sci. 2025 Jan 19;26(2):826. doi: 10.3390/ijms26020826 (PMC11765557; doi:10.3390/ijms26020826)
Supplement: Supplementary file 1 [file ijms-26-00826-s001.zip › ijms-3411349-supplementary.pdf]

Supplementary Matherials

Table S1. Multiple Regression analyses

Multiple regression analyses were performed to identify the best predictors of verbal memory. The model includes MRI characteristics, age, sex, and EDSS at diagnosis, CSF KFLC, CSF LFLC, K- Index and Lambda-Index at baseline as independent variables; CVLT normalized T-scores as dependent variables. *Abbreviations:* CSF cerebrospinal fluid, df : degrees of freedom, EDSS: expanded disability status score, Gd : gadolinium, KFLC : kappa free light chanis, LFLC : lambda free light chains, SC : spinal cord , SE: standard error, WMLL white matter lesion load.

Model Summary

				Change statistics				
R	R2	Adjusted R2	SE of the estimate	R2 change	F change	df1	df2	Sign. F change
,538[a]	,289	,147	11,060	,289	2,033	10	50	***,049***

a. Predictors: (costant), SC lesions, Gd status, WMLL, age at diagnosis, CSF KFLC, CSF LFLC, Kappa-Index, Lambda-Index sex, EDSS at diagnosis

Coefficients[a]

	Unstandardized coefficients		Standardized coefficients			95,0% CI for B	
	B	SE	Beta	t	Sign.	Lower bound	Upper bound
(Costant)	80,259	13,662		5,875	,000	52,819	107,700
Age at diagnosis	-,241	,149	-,208	-1,609	,114	-,541	,060
CSF KFLC	-17,846	6,100	-,968	-2,925	***,005***	-30,099	-5,593
CSF LFLC	,464	7,424	,010	,063	,950	-14,447	15,375
K-Index	,114	,046	,854	2,460	***,017***	,021	,207
L-Index	-,118	,082	-,247	-1,437	,157	-,284	,047
WMLL	-3,697	3,043	-,152	-1,215	,230	-9,809	2,414
GD	-,598	3,435	-,025	-,174	,862	-7,498	6,301
SC	-,746	3,181	-,029	-,235	,815	-7,136	5,643
Sex	-4,422	3,547	-,177	-1,247	,218	-11,546	2,703
EDSS	-3,057	1,720	-,239	-1,777	,082	-6,511	,398

a. dependent variable: CVLT T-Scores
